# Variability in testing policies and impact on reported *Clostridium difficile* infection rates: results from the pilot Longitudinal European *Clostridium difficile* Infection Diagnosis surveillance study (LuCID)

**DOI:** 10.1007/s10096-016-2746-1

**Published:** 2016-09-02

**Authors:** K. Davies, G. Davis, F. Barbut, C. Eckert, N. Petrosillo, M. H. Wilcox

**Affiliations:** 1 Healthcare Associated Infections Research Group, University of Leeds, Leeds, UK; 2 Microbiology, Old Medical School, Leeds General Infirmary, Leeds, LS1 3EX W. Yorks UK; 3 National Reference Laboratory for Clostridium difficile, Saint-Antoine Hospital, Paris, France; 4 National Institute for Infectious Diseases, Rome, Italy

## Abstract

Lack of standardised *Clostridium difficile* testing is a potential confounder when comparing infection rates. We used an observational, systematic, prospective large-scale sampling approach to investigate variability in *C. difficile* sampling to understand *C. difficile* infection (CDI) incidence rates. In-patient and institutional data were gathered from 60 European hospitals (across three countries). Testing methodology, testing/CDI rates and case profiles were compared between countries and institution types. The mean annual CDI rate per hospital was lowest in the UK and highest in Italy (1.5 vs. 4.7 cases/10,000 patient bed days [pbds], *p* < 0.001). The testing rate was highest in the UK compared with Italy and France (50.7/10,000 pbds vs. 31.5 and 30.3, respectively, *p* < 0.001). Only 58.4 % of diarrhoeal samples were tested for CDI across all countries. Overall, only 64 % of hospitals used recommended testing algorithms for laboratory testing. Small hospitals were significantly more likely to use standalone toxin tests (SATTs). There was an inverse correlation between hospital size and CDI testing rate. Hospitals using SATT or assays not detecting toxin reported significantly higher CDI rates than those using recommended methods, despite testing similar testing frequencies. These data are consistent with higher false-positive rates in such (non-recommended) testing scenarios. Cases in Italy and those diagnosed by SATT or methods NOT detecting toxin were significantly older. Testing occurred significantly earlier in the UK. Assessment of testing practice is paramount to the accurate interpretation and comparison of CDI rates.

## Introduction

While *Clostridium difficile* infection (CDI) is a key healthcare challenge, standardised disease measurement remains elusive [1–4]. Mandatory CDI reporting is rare, although more countries are introducing or expanding surveillance of healthcare-associated CDI [2, 3]. Guidelines recommend early detection of CDI and active surveillance as essential to disease control [5–7]. Reported CDI incidence can vary markedly between hospitals and countries; CDI rates across European countries varied ∼40-fold in 2008 (0.0–36.3/10,000 patient bed days [pbds], per hospital) and 2013 (0.7–28.7/10,000 pbds) [8, 9]. Key CDI risk factors, including age and antibiotic exposure, should be similar across countries; thus, such high variability suggests that other dominant factors influence reported CDI rates.

A recent US study, based on data from 3.6 % of the population, estimated a total of 453,000 cases in 2011 [4]. Notably, a sensitivity analysis explored the effects of testing method: the total number of cases could vary from 286,300 to 701,100 if the proportion of samples tested for CDI by polymerase chain reaction (PCR) ranged from 0 to 100 % [4]. Using single *C. difficile* targets (e.g. toxin gene) can exaggerate reported case rates, as such testing policies do not differentiate between colonisation and true CDI [10–12]. The detection of *C. difficile* toxin correlates with mortality, disease severity and complications [11, 13], but available assays are sub-optimal, as standalone toxin tests (SATTs) have sub-optimal sensitivity and specificity.

In addition to testing methodology, diagnostic intensity (mostly driven by CDI awareness) is a key determinant of reported rates [8, 14, 15]. In 2012, only ∼40 % of 482 hospitals across Europe were using recommended laboratory methods to diagnose CDI [8]. Furthermore, 23 % of all CDI-positive samples identified at study coordinating laboratories were never even tested at the submitting hospital due to a lack of clinical awareness [8]. We have used a systematic, observational prospective large-scale sampling approach to investigate variability in *C. difficile* sampling, testing and reported CDI rates.

## Methods

Sixty hospitals across France, Italy and the UK (20 per country) were recruited by national coordinators to represent a wide geographical area. Via a questionnaire, hospitals provided institutional data (size and type of institution) and details of current CDI laboratory diagnostic methods and policies (April 2014 to March 2015). We obtained monthly data for hospital in-patients on the numbers of enteropathogen and CDI tests, and CDI-positive cases, along with case demographic data. Cases were defined as ‘primary’ if the first case in the patient or ‘recurrent’ if a second positive sample occurred within 2–8 weeks of a previous positive. All data were uploaded prospectively by participating hospitals to a secure, dedicated, web-based, study database. All data analyses were conducted by the European coordinator.

### Data analysis

The testing methodology, testing/CDI rates and case profiles were compared between countries and different sized institutions. Hospitals were classified as small (<100,000 pbds per annum), medium (100,000–500,000) or large (>500,000). Annual testing and case rates were calculated per 10,000 pbds for each hospital, and mean rates were compared between countries and between different sized institutions. The mean CDI case incidence and patient profiles were compared between hospitals using a recommended testing algorithm (GDH/toxin or NAAT/toxin) versus methods not detecting toxin (e.g. GDH/NAAT or NAAT alone), SATT (e.g. toxin EIA alone) or a non-recommended algorithm (e.g. GDH/toxigenic culture) [1]. CDI and testing rates were compared by analysis of variance (ANOVA), age distributions by Kruskal–Wallis and proportions were compared by Chi-squared. Analysis was performed on SPSS 19 (IBM).

This surveillance study was granted ethical approval by the University of Leeds (SoMREC13032) for UK data collection and European-wide analysis, and by the National Institute for Infectious Diseases ‘Spallanzani’, Rome for Italian data collection. Ethical approval was not required in France.

## Results

### Institutional data

Fifty-nine of 60 hospitals completed the questionnaire (one missing in the UK). There were five small, 40 medium and 12 large hospitals; data on the number of pbds were not available for a further two hospitals. There were more small hospitals in Italy than in either France or the UK; Italy had no large hospitals (Table 1).

**Table 1:** Annual testing, *Clostridium difficile* infection (CDI) and recurrence rates/10,000 patient bed days (pbds) per hospital for each country, and for small (<100,000 pbds per annum), medium (100,000–500,000 pbds) or large (>500,000 pbds) hospitals

Country	Size of hospital	Number of hospitals (*n*)	Average number of faecal samples tested for enteropathogens/10,000 pbds per hospital per annum (*n*)	Average number of patients tested for enteropathogens /10,000 pbds per hospital per annum (*n*)	Average number of faecal samples tested for CDI/10,000 pbds per hospital per annum (*n*)	Average number of patients tested for CDI/10,000 pbds per hospital per annum (*n*)	Average number of faecal samples positive for CDI/10,000 pbds per hospital per annum (*n*)	Average number of patients positive for CDI/10,000 pbds per hospital per annum (*n*)	Number of recurrent CDI cases/10,000 pbds per hospital per annum (*n*)
France	No data	1	n/a	n/a	n/a	n/a	n/a	n/a	n/a
France	Small	0	n/a	n/a	n/a	n/a	n/a	n/a	n/a
France	Medium	13	72.3	54.8	40.7	34.3	3.5	3.2	0.4
France	Large	6	51.4	40.2	26.1	21.6	2.2	2.1	0.3
France	Overall	20	68.3	51.0	36.2	30.3	3.1	2.9	0.4
Italy	No data	0	n/a	n/a	n/a	n/a	n/a	n/a	n/a
Italy	Small	4	284.8	212.7	67.0	49.7	7.6	6.1	1.0
Italy	Medium	16	140.1	90.5	37.1	30.1	4.8	4.6	0.6
Italy	Large	0	n/a	n/a	n/a	n/a	n/a	n/a	n/a
Italy	Overall	20	150.4	99.2	39.2	31.5	5.0	4.7	0.6
UK	No data	1	n/a	n/a	n/a	n/a	n/a	n/a	n/a
UK	Small	1	139.5	114.2	103.4	90.3	3.2	3.0	0.5
UK	Medium	11	206.2	161.7	94.4	83.0	2.1	2.0	0.2
UK	Large	6	75.9	35.8	42.4	32.1	1.1	1.1	0.2
UK	Overall	19	123.9	74.1	64.2	50.7	1.6	1.5	0.2
Overall	No data	2	n/a	n/a	n/a	n/a	n/a	n/a	n/a
Overall	Small	5	262.8	197.8	72.5	55.8	7.0	5.6	0.9
Overall	Medium	40	129.2	94.6	54.1	46.2	3.5	3.3	0.4
Overall	Large	12	67.8	37.3	37.0	28.6	1.5	1.4	0.2
Overall	Overall	59	107.4	69.2	50.3	40.5	2.7	2.5	0.3

### Testing and case rates: comparison between countries and different sized institutions

The mean annual overall enteropathogen testing rate per hospital was 107.4 tests/10,000 pbds, with significantly more tests carried out in Italy than the other two countries (*p* < 0.001) (Table 1). The UK had the highest rate of CDI patient tests/10,000 pbds compared with Italy and France (50.7 vs. 31.5 and 30.3, respectively, *p* < 0.001) (Table 1). If the number of in-patient samples sent for enteropathogen detection is used as a proxy for the number of diarrhoeal samples submitted to the laboratory, then, overall, only 58.4 % of diarrhoeal samples were tested for CDI across the three countries. The UK and France tested higher proportions of in-patient diarrhoeal samples for CDI (68.5 and 59.4 %, respectively) than Italy (31.8 %).

**Fig. 1:**
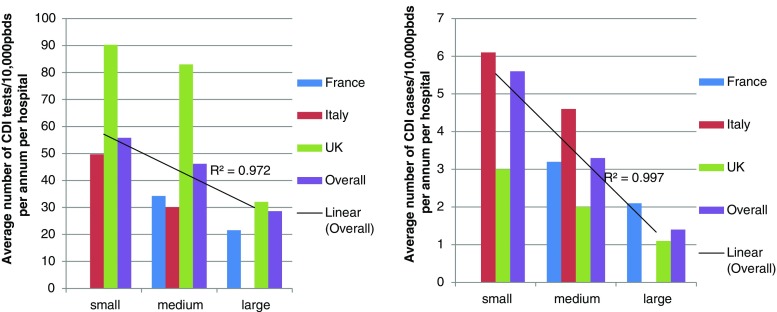
Effect of size of the hospital on *Clostridium difficile* infection (CDI) testing and case rates/10,000 patient bed days (pbds) per hospital per annum. R^2^ (linear trendline) are for overall values only. For CDI case rates, Spearman’s r = 0.135, *p* < 0.001

The mean annual CDI rate per hospital was 2.5/10,000 pbds, with the lowest incidence in the UK and the highest in Italy (1.5 vs. 4.7 cases/10,000 pbds, respectively, *p* < 0.001) (Table 1). The mean annual rate of laboratory-defined CDI recurrence per hospital was 0.3/10,000 pbds; significantly higher recurrence rates were reported in Italy than in the other two countries (Italy 0.6 vs. France 0.4 and the UK 0.2/10,000 pbds, *p* = 0.026) (Table 1). The mean annual CDI testing and CDI-positive rates were significantly higher in small hospitals (55.8/10,000 pbds and 5.6/10,000 pbds, respectively) compared with medium (46.2/10,000 pbds and 3.3/10,000 pbds, respectively) and large hospitals (28.6/10,000 pbds and 1.5/10,000 pbds, respectively) (*p* < 0.001 and *p* = 0.05) (Table 1, Fig. 1 R^2^ = 0.972 and 0.9975, respectively, *p* < 0.001). Small hospitals also had the highest annual rate of recurrence (0.9/10,000 pbds, *p* < 0.001) (Table 1), although this was largely driven by the high rate of recurrence in small hospitals in Italy (Table 1).

### Testing methodology: impact on testing and case rates

**Fig. 2:**
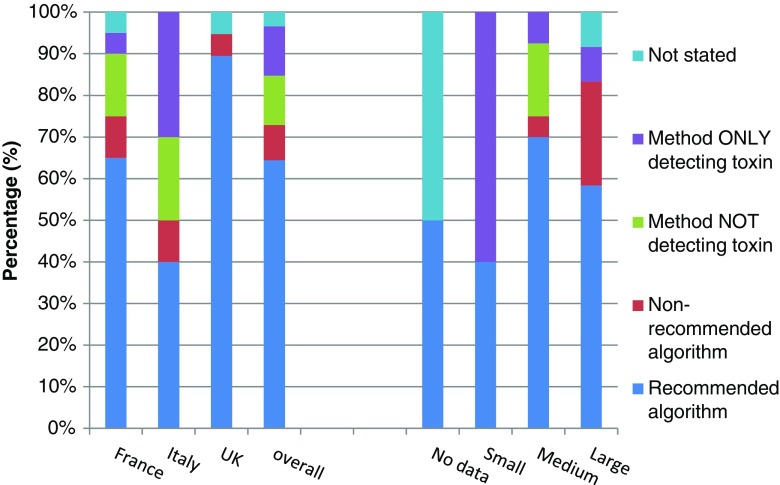
Proportion of hospitals using different CDI testing methods by country and size of institution

Overall, 38/60 (63.3 %) hospitals used a recommended testing algorithm. Significantly more hospitals in the UK (17/20, 85 %) used a recommended testing algorithm compared to those in either France (13/20, 65 %) or Italy (8/20, 40 %); both *p* = 0.001 (Table 3). Significantly more hospitals in Italy (6/20, 30 %) used SATT than institutions in either France (1/20, 5 %) or UK (0 %); both *p* = 0.004 (Fig. 2). Small hospitals (3/5, 60 %) were significantly more likely to use SATT than medium (3/40, 8 %) or large (1/12, 8 %) hospitals; both *p* = 0.005 (Fig. 2).

The mean annual hospital-reported CDI rate was significantly higher for those institutions using methods that do not detect toxin or SATT (5.2 and 4.0/10,000 pbds, respectively) versus hospitals using recommended algorithms (2.0/10,000 pbds; *p* < 0.001) (Table 3), despite testing rates being similar. The CDI positivity rate was 2.5-fold higher when methods not detecting toxin versus a recommended algorithm were used (14.1 % vs. 5.4 %, *p* < 0.001) and 3.5-fold higher for SATT (18.6 vs. 5.4 %, *p* < 0.001). In addition, hospitals using methods that do not detect toxin or SATT had (non-significantly) higher mean annual CDI recurrence rates (0.7 and 0.4/10,000 pbds, respectively) than those using a recommended algorithm (0.3/10,000 pbds).

### CDI case demographics

Between April 2014 and March 2015, there were 5876 reported CDI cases, of which 4937 (84 %) were first episodes (data unavailable for 176). Of 5855 cases with gender data available, there were slightly more females with CDI (3252, 55 %). There was a significant difference in the gender of cases between countries; proportion of females, France 53.3 %, Italy 58 % and the UK 56.5 % (*p* = 0.009) (Table 2). The median age of CDI cases was 75 years [interquartile range (IQR) 60–84, range 0–104], but 30 % were aged <65 years. There was a significant difference in the median age and distribution of ages of cases between countries; CDI patients in Italy were older than those in France and the UK (*p* < 0.001) (Table 2). Additionally, the median ages of cases diagnosed in hospitals using methods either not detecting toxin (77 years) or SATT (81 years) were significantly older than those tested in hospitals using the recommended algorithm (75 years, both *p* < 0.001). Also, patients with recurrent CDI were significantly older than those with primary infection (76 vs. 74 years, *p* = 0.007).

**Table 2:** Demographics of CDI-positive cases from each country and overall

Country	France	Italy	UK	Overall
Gender				
No. of females/total	1334/2501	930/1604	988/1750	3252/5855
(% females)	(53.3)	(58.0)	(56.5)	(55.5)
Median age (years)	71	78	74	75
(IQR, range)	(55–83, range 0–104)	(67–85, range 0–101)	(61–84, range 0–104)	(60–84, range 0–104)
Specialty location of patient, *n* (%)				
Medical	1895 (75.6)	1277 (78.3)	1201 (67.4)	4373 (73.9)
Surgical	227 (9.1)	143 (8.8)	380 (21.3)	750 (12.7)
ITU/HDU	261 (10.4)	105 (6.4)	91 (5.1)	457 (7.7)
Obstetrics/gynaecology	11 (0.4)	24 (1.5)	40 (2.2)	75 (1.3)
Paediatric	111 (4.4)	24 (1.5)	40 (2.2)	175 (3.0)
No data	2 (0.1)	58 (3.6)	29 (1.6)	89 (1.5)
Total	2507	1631	1781	5919

Specialty data were available for 5919 CDI cases; 73.9 % were in medical specialities, 12.7 % surgery, 7.7 % ITU/HDU, 1.3 % obstetrics/gynaecology and 3.0 % paediatrics (Table 2). There were more UK surgical patients than in France or Italy (*p* < 0.001), and more ITU/HDU patients in France than in Italy or the UK (*p* < 0.001).

### Time from admission to testing

UK cases were tested significantly earlier (median day 3, IQR 0–13, range 0–520; *p* < 0.001) than in France (median day 6, 1–22, 0–3874) or Italy (median day 10, 3–20, 1–414). Almost half (48.9 %) of UK CDI-positive samples were tested ≤48 h after admission, compared with 27.5 and 34.3 % in Italy and France, respectively.

## Discussion

This pilot study demonstrates the feasibility of collecting and comparing prospective data on CDI testing across hospitals and countries. We have clearly shown the impacts of testing policies and methodologies on the reported rates of CDI and case demographics. There are three main drivers of CDI rates that can distort true incidence: diarrhoea sampling frequency; rate of CDI testing (testing/requesting frequency) and laboratory method(s). A European study in 2008 showed a close correlation between testing frequency and reported CDI rate [8]. Five years later, albeit using a considerably larger hospital cohort, this association was much weaker, likely reflecting greater heterogeneity of testing methods [8].

Targeted testing of ‘at-risk’ patients will also likely impact on CDI rates. CDI patients in Italy were significantly older than those in the other two countries. Either CDI cases in Italy are genuinely older or, more likely, *C. difficile* testing is generally performed on older patients. Although older age is often reported as a risk factor for CDI, this may, in fact, be a proxy marker for co-morbidity risk [16]. We have shown previously that CDI under-diagnosis is more likely to occur in younger individuals, presumably due to the lack of clinical suspicion and/or testing policies that give insufficient prominence to (real or perceived) lower risk groups [8]. In the present study, patients diagnosed by non-recommended methods were significantly older than those identified by recommended testing algorithms. It appears, therefore, that targeted testing and the use of non-recommended testing methods may be linked.

Interestingly, whilst UK hospitals appear not to be targeting *C. difficile* testing in the elderly, there is a clear bias towards earlier sampling/testing, compared with practice in institutions in either France or Italy; CDI cases were tested a median of 3–7 days earlier in the former (median 3 vs. 6 and 10 days, respectively; *p* < 0.001). Earlier CDI testing in the UK likely reflects the desire to apportion cases as ‘community-’ (diagnosed before or within the first 2 days of hospital admission) rather than ‘hospital-acquired’ (diagnosed after 2 days of hospital admission); notably, there are financial penalties associated with excess ‘hospital-acquired’ CDI cases in the UK. Earlier testing is also likely to be partly driven by differing country policies for sample submission; in France and Italy, sampling is recommended after 48 h of diarrhoea, whereas in the UK, this can occur after only one diarrhoeal episode [17]. It is possible that such earlier testing will result in higher reported CDI rates, as some cases would have resolved without treatment.

Sub-optimal CDI diagnosis is still prevalent within Europe [8, 14, 15], despite guidelines having been issued in 2009 [1]. Only 64 % of the 60 hospitals in the present study used recommended laboratory testing algorithms [1]. More UK hospitals used recommended algorithms for CDI diagnosis (85 % vs. 40–65 %), reflecting performance management of national guidelines that were issued in 2012 [17]; indeed, the UK rate of ‘recommended’ testing has increased since 2013 (76 %) [8]. According to our data, almost a third of Italian hospitals are still using SATT for CDI diagnosis, despite clear evidence of their poor prognostic performance (i.e. sub-optimal positive and negative predictive values) [18, 19]. Small hospitals were significantly more likely to use SATT, although all 3/5 such hospitals were in Italy; thus, this association may be a country effect and/or related to institutional size. There was, however, a significant inverse relationship between hospital size and both *C. difficile* testing rate and CDI case frequency (Fig. 1). It is likely that high CDI rates in small hospitals reflect more testing. However, it is possible that there is also some confounding here, as the use of SATT is associated with a high false-positive CDI rate [11, 18, 19]. In addition, smaller hospitals may have a different patient and ward mix to that seen in larger institutions; this again may impact on CDI rates.

In hospitals using diagnostic methods that only detected toxin or did not detect toxin at all, CDI rates were significantly higher than in institutions using recommended algorithms, despite testing rates being similar (Table 3). It could be argued that assays not detecting toxin (including standalone NAAT testing) are more sensitive and, thus, are detecting more cases, which are missed by other methods. However, several studies have shown that diarrhoeal patients that are *C. difficile* NAAT-positive only do not have higher mortality or CDI complications than controls, in contrast to cases defined by the presence of faecal *C. difficile* toxins [10–13]. It is likely, therefore, that the higher case rates seen in hospitals not using toxin detection are due to the detection of colonised patients in addition to those with true CDI.

**Table 3:** Annual testing, CDI and recurrence rates/10,000 pbds per hospital for each country, for different testing methodologies; Recommended algorithms (GDH/toxin or NAAT/toxin); non-recommended algorithms (e.g. culture/toxin detection); methods NOT detecting toxin (e.g. NAAT alone) or methods ONLY detecting toxin (e.g. standalone toxin EIA)

Country	Testing method	Number of hospitals (*n*)	Average number of faecal samples tested for enteropathogens/10,000 pbds per hospital per annum (*n*)	Average number of patients tested for enteropathogens /10,000 pbds per hospital per annum (*n*)	Average number of faecal samples tested for CDI/10,000 pbds per hospital per annum (*n*)	Average number of patients tested for CDI/10,000 pbds per hospital per annum (*n*)	Average number of faecal samples positive for CDI/10,000 pbds per hospital per annum (*n*)	Average number of patients positive for CDI/10,000 pbds per hospital per annum (*n*)	Number of recurrent CDI cases/10,000 pbds per hospital per annum (*n*)
France	Recommended algorithm	13	54.4	42.1	32.4	27.5	2.6	2.4	0.4
France	Methods NOT detecting toxin	3	102.4	71.4	49.3	40.1	5.0	4.5	0.4
France	Non-recommended algorithm	2	72.3	56.8	30.9	28.0	2.3	2.2	0.2
France	Methods ONLY detecting toxin	1	33.2	33.2	21.6	11.3	3.0	3.0	0.2
France	Not stated	1							
Italy	Recommended algorithm	8	114.3	70.9	38.0	30.8	5.1	4.7	0.7
Italy	Methods NOT detecting toxin	4	294.9	170.7	41.4	31.0	6.8	6.5	1.1
Italy	Non-recommended algorithm	2	135.8	104.9	39.9	33.4	2.2	2.2	0.0
Italy	Methods ONLY detecting toxin	6	121.3	106.0	40.1	32.5	5.7	5.1	0.6
Italy	Not stated	0							
UK	Recommended algorithm	17	104.2	66.2	51.4	42.2	1.3	1.3	0.2
UK	Methods NOT detecting toxin	0							
UK	Non-recommended algorithm	1	156.6	112.7	141.1	103.1	3.2	2.7	0.3
UK	Methods ONLY detecting toxin	0							
UK	Not stated	1	420.5	167.1	200.2	140.2	5.4	4.3	1.4
Overall	Recommended algorithm	38	89.6	59.1	44.1	36.5	2.1	2.0	0.3
Overall	Methods NOT detecting toxin	7	171.0	106.8	46.5	36.9	5.6	5.2	0.7
Overall	Non-recommended algorithm	5	105.2	79.8	59.3	47.3	2.5	2.3	0.2
Overall	Methods ONLY detecting toxin	7	75.6	68.2	30.5	21.5	4.3	4.0	0.4
Overall	Not stated	2	508.8	214.0	238.6	171.6	9.0	7.7	2.1

There are several limitations to this pilot study. Firstly, this was an observation study that collected data at the laboratory level, with no information available on symptom severity; we presumed that diarrhoea was present, based on assumed clinical decisions to sample and/or request *C. difficile* testing. Institutions that participated in this study were chosen by the study coordinators and may have had a higher CDI awareness and/or rates; however, institutional selection was intended to be ‘representative’ of hospitals nationally, and to cover a wide geographical spread. We did not collect demographic data for patients with samples that tested negative for *C. difficile*/CDI. This would provide better evidence for the age distribution of cases/controls and help to show how testing is targeted. Such data will be collected in the follow-on full LuCID study, which will also expand to include a total of 40 hospitals in each country and two more countries (Spain and Germany). The additional countries and hospitals will increase the sizes of the (at present) small sub-groups, such as small hospitals and CDI recurrences. As patient bed day data were collected annually rather than per month, we were not able to determine with confidence possible seasonality of either CDI testing or case rates. These limitations will also be addressed in the full LuCID study.

Our pilot results show that it is important to understand the context of sampling, testing and methodology in order to interpret reported CDI rates. It is essential that studies either take such potential confounders into account when interpreting data, and, ideally, that large, multi-centre, multi-country studies are used to determine the true epidemiology of CDI. Additionally, we have shown that inclusive testing with recommended diagnostic methodologies is associated with lower reported rates of CDI. Theoretically, increased awareness of true CDI cases enhances opportunities to implement appropriate and targeted infection prevention and control measures. ‘Missed’ cases, either through lack of clinical suspicion or the use of non-recommended laboratory diagnostics, may facilitate the transmission of *C. difficile* because of unrecognised reservoirs of infection. Conversely, false-positive cases may be receiving antibiotics for assumed CDI that could be detrimental, potentially having deleterious or resistance selection effects on gut microbiota and possible induction of true CDI in some instances.
